# The transcription factor TCFL5 responds to A-MYB to elaborate the male meiotic program in mice

**DOI:** 10.1530/REP-22-0355

**Published:** 2023-01-04

**Authors:** Katharine Cecchini, Adriano Biasini, Tianxiong Yu, Martin Säflund, Haiwei Mou, Amena Arif, Atiyeh Eghbali, Cansu Colpan, Ildar Gainetdinov, Dirk G. de Rooij, Zhiping Weng, Phillip D. Zamore, Deniz M. Özata

**Affiliations:** 1 RNA Therapeutics Institute and Howard Hughes Medical Institute, University of Massachusetts Medical School, 368 Plantation Street, Worcester, MA 01605, USA; 2 Program in Bioinformatics and Integrative Biology, University of Massachusetts Medical School, Worcester, MA 01605, USA; 3 Department of Molecular Biosciences, The Wenner-Gren Institute, Stockholm University, S-106 91 Stockholm, Sweden; 4 Cold Spring Harbor Laboratory, Cold Spring Harbor, NY 11724, USA; 5 Present address: Beam Therapeutics, 238 Main St, Cambridge, MA 02142, USA; 6 Present address: Voyager Therapeutics, 75 Sidney St, Cambridge, MA 02139, USA; 7 Reproductive Biology Group, Division of Developmental Biology, Department of Biology, Faculty of Science, Utrecht University, Utrecht 3584, the Netherlands; 8 Lead contact

**Keywords:** TCFL5, spermatogenesis, meiosis, A-MYB, transcription factors network, miR-34/449

## Abstract

In male mice, the transcription factors STRA8 and MEISON initiate meiosis I. We report that STRA8/MEISON activates the transcription factors A-MYB and TCFL5, which together reprogram gene expression after spermatogonia enter into meiosis. TCFL5 promotes transcription of genes required for meiosis, mRNA turnover, miR-34/449 production, meiotic exit, and spermiogenesis. This transcriptional architecture is conserved in rhesus macaque, suggesting TCFL5 plays a central role in meiosis and spermiogenesis in placental mammals. *Tcfl5*^*em1/em1*^ mutants are sterile, and spermatogenesis arrests at the mid- or late-pachytene stage of meiosis. Moreover, *Tcfl5*^*+/em1*^ mutants produce fewer motile sperm.

## In brief

The testis-specific transcription factor, TCFL5, expressed in pachytene spermatocytes regulates the meiotic gene expression program in collaboration with the transcription factor A-MYB.

## Introduction

Gametogenesis converts diploid progenitor germ cells into haploid gametes specialized for sexual reproduction. Male gametogenesis encompasses a stepwise developmental pathway of meiotic cell divisions and differentiation to generate mature sperm from self-renewing spermatogenic stem cells (Handel and Schimenti, 2010). Because haploid sperm are mostly transcriptionally inert (Grunewald et al., 2005; Kierszenbaum and Tres, 1975; Ostermeier et al., 2002), meiotic cells must express not only gene products required for the orderly progression and completion of meiosis, but also produce the transcripts encoding proteins required to build mature sperm (Bolcun-Filas et al., 2011; Handel and Schimenti, 2010).

A-MYB binds promoters, super enhancers, and endogenous retroviral enhancers that promote transcription of meiotic genes (Maezawa et al., 2020; Sakashita et al., 2020). Although *A-Myb*-mutant germ cells fail to express meiotic genes and arrest early in meiosis I (Bolcun-Filas et al., 2011), A-MYB directly binds the promoters of less than one-quarter of meiotic genes (Bolcun-Filas et al., 2011; Li et al., 2013), suggesting that one or more additional transcription factors act downstream or in parallel to A-MYB to regulate meiotic gene transcription.

Meiotic distal super enhancers often contain the DNA-binding motif of the testis-specific transcription factor TCFL5 (Maezawa et al., 2020), and *Tcfl5* homozygous and heterozygous mutant mice are male sterile (Galán-Martínez et al., 2022; Xu et al., 2022). Here, we report that, in response to retinoic acid (RA), STRA8/MEIOSIN initiate the transcription of *A-Myb* and *Tcfl5*. TCFL5 promotes the transcription of genes required for meiosis, meiotic exit, as well as genes related to spermiogenesis. Moreover, the A-MYB/TCFL5 regulatory architecture establishes a coherent feedforward loop to ensure transcription of miR-34/449—a family of miRNAs with essential roles in spermatogenesis (Comazzetto et al., 2014)—during male meiosis I. Without TCFL5, male mouse germ cells arrest at the mid- or late-pachynema. This transcriptional architecture is evolutionarily conserved in the old-world monkey rhesus macaque, suggesting it predates the divergence of rodents and primates. Our findings identify a transcriptional circuit that regulates meiotic gene expression.

## Materials and methods

### Mice

Mice were maintained and used according to the guidelines of the Institutional Animal Care and Use Committee of the University of Massachusetts Medical School (A201900331). The committee specifically approved this study. C57BL/6J mice (RRID: IMSR_JAX:000664) were used as wild-type controls. *A-Myb*^*repro9*^ (MGI3512907) were a gift from John Schimenti, and were maintained in a C57BL/6 background.

*Tcfl5*^*em1/em1*^ mutant mice was generated and their genotypes were screened as described in Yu et al. (2022). Briefly, two sgRNAs (20 ng/μl each) targeting sequences in *Tcfl5* intron 1 (5′-GCA GUC UGG GUA CUA GAUA G-3′) and intron 3 (5′-AUU CAC UCA AAC AAC AAG AG-3′) and Cas9 mRNA (50 ng/μl, TriLink Biotechnologies, L-7206) were injected into the pronucleus and cytoplasm of fertilized eggs (Transgenic Animal Modeling Core, University of Massachusetts Medical School), and 15–25 blastocysts were transferred into uterus of pseudo-pregnant ICR females at day E2.5. *Tcfl5*^*+/FLAG*^ was generated as described in Yu et al. (2022).

### Isolation of mouse germ cells by FACS

Germ cell sorting was as described (Gainetdinov et al., 2018; Yu et al., 2022). Briefly, we de-capsulated the testis specimens and incubated with 1× Gey′s Balanced Salt Solution (GBSS, Sigma, G9779) containing 0.4 mg/ml collagenase type 4 (Worthington; LS004188) at 33°C for 15 min. Thereafter, we washed the seminiferous tubules twice with 1× GBSS, and incubated them with 1× GBSS containing 0.5 mg/ml trypsin and 1 μg/ml DNase I at 33°C for 15 min. Next, the seminiferous tubules were gently pipetted up and down for 3 min through a Pasteur pipette to homogenize at 4°C on. Trypsin was inactivated with fetal bovine serum (FBS; f.c. 7.5% [v/v]), and the cell suspension was passed through a pre-wetted 70 μm cell strainer. Cells were recovered by centrifugation at 300 × *g* at 4°C for 10 min, resuspended in 1× GBSS containing 5% (v/v) FBS, 1 μg/ml DNase I, and 5 μg/ml Hoechst 33342 (Thermo Fisher, 62249), and incubated at 33°C for 45 min rotating at 150 rpm. Finally, we added propidium iodide (0.2 μg/ml, f.c.; Thermo Fisher, P3566) directly to the cells which were further filtered through a pre-wetted 40 μm cell strainer. Cell sorting (FACS Core, University of Massachusetts Medical School) was performed as described (Bastos et al., 2005; Yu et al., 2022).

### Testis histology

Wild-type and *Tcfl5*^*em1/em1*^ mutant testis tissue was fixed with Bouin’s solution, and paraffin embedded tissue sectioned at 5 μm thickness, and then stained with hematoxylin and eosin (H&E) solutions (Morphology Core Facility, University of Massachusetts Medical School).

### Sperm motility

Cauda epidydimal sperm were incubated in warm EmbryoMax HTF media at 37°C, 5% CO2. A drop of sperm was removed from the suspension and pipetted into a sperm counting glass chamber, then assayed by CASA or video acquisition. CASA was conducted using an IVOS II instrument (Hamilton Thorne, Beverly, MA) with the following settings: 100 frames acquired at 60 Hz; minimal contrast = 50; 4-pixel minimal cell size; minimal static contrast = 5; 0%straightness (STR) threshold; 10 μm/s VAP Cutoff; prog. min VAP, 20 μm/s; 10 μm/s VSL Cutoff; 5-pixel cell size; cell intensity = 90; static head size = 0.30–2.69; static head intensity = 0.10–1.75; static elongation = 10–94; slow cells motile = yes; 0.68 magnification; LED illumination intensity = 3000; IDENT illumination intensity = 3603; 37°C. The raw data files (i.e., .dbt files for motile sperm and .dbx files for static sperm) containing tracks ≥ 45 frames were used for sperm motility analysis. CASAnova was utilized to determine fractions of progressive and hyperactivated sperm from .dbt files of motile sperm, as previously described (Goodson et al., 2011)

### Immunofluorescence

Immunofluorescent (IF) staining of FACS-purified germ cells was as described (Gainetdinov et al., 2018). Briefly, freshly sorted germ cells were incubated in 25 mM sucrose solution at room temperature for 20 min and fixed with 1% (w/v) formaldehyde solution containing 0.15% (v/v) Triton X-100 at room temperature for 2 h. After washing the slides with (i) 1 × PBS containing 0.4% (v/v) Photo-Flo 200 (Kodak, 1464510), (ii) 1× PBS containing 0.1% (v/v) Triton X-100, and (iii) 1× PBS containing 0.3% (w/v) BSA, 1% (v/v) donkey serum (Sigma, D9663), and 0.05% (v/v) Triton X-100 (10 min each wash), slides were incubated with Rabbit polyclonal anti-SYCP3 (Abcam, ab15093; 1:1,000 dilution) and mouse monoclonal anti-γH2AX (Millipore, 05–636; 1:1,000 dilution) primary antibodies in 1 × PBS containing 3% (w/v) BSA, 10% (v/v) donkey serum, and 0.5% (v/v) Triton X-100 in a humidifying chamber at room temperature overnight. Next, slides were washed sequentially as (i–iii, above), then incubated with donkey anti-rabbit IgG (H+L) Alexa Flour 488 (Thermo Fisher Scientific, A-21206; 1:2,000) and anti-mouse IgG (H+L) Alexa Flour 594 (Thermo Fisher Scientific, A-21203; 1:2,000) secondary antibodies at room temperature for 1 h. The slides were washed three times with 1 × PBS containing 0.4% (v/v) Photo-Flo 200 (10 min each wash) and once with 0.4% (v/v) Photo-Flo 200 for 10 min. Immunofluorescent images were captured using Leica SP8 with 63×, 1.4 NA oil immersion objective with post-acquisition deconvolution.

For IF staining of cryopreserved testis sections, slides were fixed with 1 × PBS containing 0.1% (w/v) sodium periodate (NaIO_4_), 3% (w/v) l-lysine and 3% (v/v) paraformaldehyde at room temperature for 20 min. Slides were blocked with 2.5% goat serum. Next, slides were incubated with rabbit polyclonal anti-SYCP3 (Abcam, ab15093; 1:300 dilution) and mouse monoclonal anti-γH2AX (Millipore, 05–636; 1:300 dilution) antibodies at 4°C overnight. After primary antibody incubation, slides were washed twice with 1 × PBS containing 0.05% (v/v) Tween-20 and incubated with goat anti-rabbit IgG (H+L) Alexa Flour 568 (Thermo Fisher Scientific, A-11011; 1:500 dilution) and goat anti-mouse IgG (H+L) Alexa Flour 488 (Thermo Fisher Scientific, A-32723; 1:500 dilution) secondary antibodies at room temperature for 2 h. Next, nuclei were counterstained with 1 mg/ml DAPI (Thermo Fisher Scientific, 62248), the slides were sealed using gold antifade mountant solution (Fisher scientific, P36930) and covered with a #1 coverslip. Immunofluorescent images were captured using a Leica DMi8 Leica microscope equipped with a 63×, 1.4 NA oil immersion objective.

### TUNEL histochemical staining

Click-iT TUNEL Colorimetric IHC Detection kit (Thermo Fisher, C10625) was used to detect DNA breaks according to the manufacturer’s protocol. In brief, Bouin solution-fixed, paraffin-embedded slides were de-paraffinized in three changes of xylene for 5 min each, gradually re-hydrated in 100% (v/v), 95% (v/v), and 70% (v/v) ethanol for 5 min each, and then washed in 1× PBS for 5 min. After pre-treating the slides with 20 μg/ml Proteinase K at room temperature for 15 min, slides were washed with water twice (2 min each). Positive control slides were treated with 0.5 U Turbo DNase (Thermo Fisher, AM2238) at room temperature for 30 min. Slides were then incubated with TdT reaction buffer (component J) containing terminal deoxynucleotidyl transferase (component L) in a humidified chamber at 37°C for 1 h. The reaction was quenched with 2x SSC for 15 min, then washed twice in PBS. Peroxidase activity was quenched in 3% (v/v) H_2_O_2_ at room temperature for 5 min. Slides were incubated with biotin azide and copper sulfate in a humidified chamber at 37°C for 30 min, then stained with peroxidase substrate at room temperature for 10 min. Nuclei were counterstained with methyl green, and the slides sealed with EcoMount (Biocare Medical, EM897L). Images were captured using Leica DMi8 microscope equipped with a 20× objective.

### Western blotting

Frozen testis tissues were homogenized in a Dounce homogenizer using 20 strokes of pestle B in RIPA lysis buffer (25 mM Tris-HCl, pH 7.6, 150 mM NaCl, 1% (v/v) NP-40, 1% sodium deoxycholate, and 0.1% (w/v) SDS) containing 1× homemade protease inhibitor cocktail (1 mM 4-(2-Aminoethyl)benzenesulfonyl fluoride hydrochloride (Sigma; A8456), 0.3 μm Aprotinin, 40 μm Betanin hydrochloride, 10 μm. E-64 (Sigma; E3132), 10 μm Leupeptin hemisulfate). Lysed tissue was then sonicated (Branson Digital Sonifier; 450 Cell Disruptor) to break nuclei. After sonication, the samples were centrifuged at 20,000 × *g* at 4°C for 30 min. The supernatant was transferred to a new tube, and protein concentration measured using the Pierce BCA Protein Assay Kit (ThermoFisher; 23225). Total protein (50 μg) from each sample was mixed with 1/4 volume of loading dye (106 mM Tris-HCl, pH 6.8, 141 mM Tris base, 2% SDS, 10% v/v glycerol, 0.51 mM EDTA, 0.22 mM SERVA Blue G and 0.175 mM Phenol Red) containing 0.2 M dithiothreitol and heated at 95°C for 6 min to denature proteins. Samples were resolved by electrophoresis through a 4–20% polyacrylamide gradient SDS gel (Thermo Fisher, XP04205BOX), and the gel transferred to PVDF membrane (Millipore, IPVH00010). The membrane was blocked with Blocking Buffer (Rockland Immunochemicals, MB-070) at room temperature for 1.5 h and incubated with rabbit polyclonal anti-A-MYB (Sigma, HPA008791; 1:1,000 dilution), rabbit polyclonal anti-MILI (Abcam, ab36764; 1:1,000 dilution), or mouse monoclonal anti-FLAG (Sigma, F3165; 1:1,000) antibody at 4°C overnight. Next, the membrane was washed three times (30 min each) with 1× PBS-T (0.1% (v/v) Tween-20 in 1× PBS) at room temperature; incubated with secondary donkey anti-rabbit IRDye 680RD antibody (LI-COR, 926-68073; 1:15,000 dilution) at room temperature for 30 min; washed three times (30 min each) with 1× PBS-T at room temperature, and signal detected using the Odyssey Infrared Imaging System (LI-COR). As a loading control, membrane was incubated with mouse anti-ACTIN antibody (Santa Cruz Biotechnology, sc-47778; 1:10,000 dilution) at room temperature for 2 h, followed by goat anti-mouse IRDye 800CW secondary antibody (LI-COR, 926-32210; 1:15,000 dilution) as described above.

### Global-run-on sequencing

#### Nuclei isolation

One and a half million FACS-purified primary spermatocytes were pelleted by centrifuging at 400g for 10 min at 4°C and gently resuspended in 1 ml swelling buffer (10 mM Tris-HCl pH 7.5 (w/v), 2 mM MgCl2 (w/v), 3 mM CaCl2 (w/v)). We added additional 9 ml swelling buffer dropwise to the resuspended cells and swirled gently to mix further and incubated on ice for five minutes. Cells were subsequently centrifuged at 400g for 10 min at 4°C, the supernatant was removed, and the cell pellet was gently resuspended in 500 μl prechilled lysis buffer 1 (9 mM Tris-HCl pH 7.5 (w/v), 1.8 mM MgCl_2_ (w/v), 2.7 mM CaCl_2_ (w/v), 10% Glycerol (v/v), 1x protease inhibitor cocktail (PIC), 0.4 U/μl RNAsIn Promega N2615). 500 μl prechilled lysis buffer 2 (9 mM Tris-HCl pH 7.5 (w/v), 1.8 mM MgCl2 (w/v), 2.7 mM CaCl2 (w/v), 10% Glycerol (v/v), 10% Igepal CA-360 (v/v), 1x PIC, 0.4 U/μl RNAsIn Promega N2615) were added dropwise and the mix was incubated for 5 min on ice. 9 ml prechilled lysis buffer 3 (9 mM Tris-HCl pH 7.5 (w/v), 1.8 mM MgCl2 (w/v), 2.7 mM CaCl2 (w/v), 10% Glycerol (v/v), 0.005% Igepal CA-360 (v/v), 1x PIC, 0.4 U/μl RNAsIn Promega N2615) was added, dropwise, to the lysate and the tube was gently swirled to mix. The mixed lysate was centrifuged at 600g for 5 min at 4°C and the supernatant was removed. The nuclei-containing pellet was resuspended in 1 ml prechilled Lysis Buffer 3 and additional 9 ml prechilled lysis Buffer 3 were added to the mixture dropwise. The nuclei were centrifuged at 600g for 5 min at 4°C, the supernatant was removed and the pellet was resuspended in 1 ml of freezing buffer (50 mM Tris-HCl pH 8.0 (w/v), 5mM MgCl2 (w/v), 0.1 mM EDTA pH 8.0 (w/v), 40% Glycerol (v/v), 1x PIC, 0.4 U/μl RNAsIn Promega N2615) and transferred to a fresh 1.7 ml Eppendorf tube. The isolated nuclei were centrifuged at 900g for 6 min at 4°C, the supernatant was removed and the pellet was resuspended in 100 μl freezing buffer. Isolated nuclei were flash frozen in liquid nitrogen and stored at −80°C until the Run-On was performed.

#### Nuclear Run-On

100 μl previously frozen isolated nuclei were thawed for 5 min on ice, mixed with 100 μl 2x Nuclear Run-On (NRO) reaction buffer (10 mM Tris-HCl pH 8.0, 5 mM MgCl2, 1 mM DTT, 300 mM KCl, 0.5 mM ATP, 0.5 mM GTP, 0.5 mM CTP, 0.5 mM Br-UTP, 1% Lauroylsarcosine sodium salt solution (Sigma; L7414), 1U/μl RNAsIn (Promega; N2615), 1x PIC) by pipetting gently and the mixture was immediately incubated at 30°C for 30 min. 24 μl 10x TURBO DNase buffer and 10 μl TURBO DNase (Thermo Fisher Scientific; AM2238) were mixed with the NRO reaction by pipetting and the mixture was incubated at 37°C for 20 min. RNA was extracted with 1 ml Trizol reagent (Life Technologies; 15596026), following the manufacturer’s instructions, precipitated with isopropanol and washed with 80% Ethanol before resuspending in 30 μl water. RNA was quantified using the Qubit RNA HS assay kit (Thermo Fisher Scientific; Q32855).

#### Br-UTP Immunoprecipitation.

50 μl Dynabeads MyOne Streptavidin T1 beads were washed by incubating with 1 ml IP buffer (150 mM NaCl, 50 mM Tris-HCl pH 8.0, 0.05% Tween-20, 1 mM EDTA, 1 mM DTT, 1x PIC, 1U/μl RNAsIn Promega N2615) at room temperature for 5 min using end-over-end tumbler. The wash solution was removed, and the beads were blocked by incubating with 300 μl blocking buffer (150 mM NaCl, 50 mM Tris-HCl pH 8.0, 0.05% Tween-20, 1 mM EDTA, 1 mM DTT, 0.1% Polyvinylpyrrolidone) for 1 h using end-over-end tumbler. Subsequently the blocking solution was removed, and the beads were kept at 4°C until required for purification.

30 μl RNA previously precipitated from the NRO reaction was incubated at 65°C for 5 min and immediately placed on ice for 3 min. Additional RNAsIn was added to 250 μl of IP buffer (final concentration 1U/μl) containing 0.2ug/μl of anti-BrdU biotin-conjugated antibody (Sigma; MAB3262B). The RNA was coupled to the antibody by rotating in the solution for 1 h at 4°C and subsequently added to the previously blocked beads. Coupling of blocked beads with RNA-antibody complex was performed by incubating at 4°C for 30 min using end-over-end tumbler. The antibody and RNA-coupled beads were washed with 500 μl IP buffer 5 times for 5 min each. Following the removal of last wash, RNA was extracted using Trizol reagent (Life Technologies; 15596026) precipitated in 10 μl water. RNA library preparation was performed as in “Long RNA library construction” below.

### Long RNA library construction and analysis

We extracted total RNA from frozen testis samples and from sorted germ cells using mirVana miRNA isolation kit (Thermo Fisher, AM1560). To quantify absolute molecule of transcripts, we added 1 μl of 1:100 dilution of ERCC spike-in mix 1 (Thermo Fisher, 4456740, LOT00418382) to 0.5–1μg total RNA, before RNA library construction from FACS-purified germ cells. Library construction was performed as described (Yu et al., 2022; Özata et al., 2020) and sequenced as 79 + 79 nt paired-end reads using a NextSeq500 (Illumina).

RNA-seq analysis is performed as described in ref. Yu et al. (2022). Transcript abundance was reported as reads per million uniquely mapped reads per thousand nucleotides (RPKM) calculated using homemade Bash scripts.

Transcript abundance in molecules per cell was performed as described (Gainetdinov et al., 2018). Because each sample contained ~623,291,645 molecules of ERCC spike-in mix, the abundance of each gene = (number of mapped reads × 623291645)/(number of cells used to prepare the library × the number of reads mapping to the ERCC spike-in sequences). RNA sequencing statistics are provided in Table S1. All analyses used Ensembl-v86 gene annotations.

### Chromatin immunoprecipitation and sequencing

Chromatin Immunoprecipitation (ChIP) was performed as described (Li et al., 2013; Yu et al., 2022; Özata et al., 2020). Briefly, we minced the frozen testis tissue on dry ice and fixed them in 1.7 ml tubes in ice-cold PBS containing 2% (w/v) formaldehyde at room temperature for 30 min using tumbling end-over-end. Fixed tissue was crushed in ChIP lysis buffer (1% SDS, 10 mM EDTA, 50 mM Tris-HCl, pH 8.1) with a Dounce homogenizer using 40 strokes pestle B (Kimble-Chase, Vineland, USA). We sonicated the samples to shear chromatin to 150–200 bp (Covaris, E220). Sonicated lysate was then diluted 1:10 with ChIP dilution buffer (0.01% SDS, 1.1% Triton X-100, 1.2 mM EDTA, 16.7 mM Tris-HCl, pH 8.1, 167 mM NaCl) and immunoprecipitated using 5 μg anti-TCFL5 (Sigma, HPA055223, lot # 000000907) or 5.5 μg anti-A-MYB (Sigma, HPA008791, lot # C105987) antibody. After immunoprecipitation, DNA was extracted with phenol:chloroform:isoamyl alcohol (25:24:1; pH 8), and ChIP-seq libraries generated as described (Yu et al., 2022; Özata et al., 2020). Libraries were sequenced as 79 + 79 nt paired-end reads using a NextSeq500 (Illumina).

ChIP-seq analysis using MACS 1.1.2 (ref. Zhang et al. (2008)) with parameters -keep-dup all -q 0.001 was performed as described in refs. (Yu et al., 2021; Yu et al., 2022; Özata et al., 2020). ChIP sequencing statistics are provided in Table S1.

The distance (bp) of transcription start site to the nearest TCFL5 or A-MYB peak summit for each gene was calculated. Genes that retain TCFL5 or A-MYB peak within the range of 500 bp around their transcription start site were considered as TCFL5- or A-MYB-regulated genes.

### CUT&RUN Sequencing

Cleavage Under Targets and Release Using Nuclease (CUT&RUN) sequencing from FACS-purified germ cells was performed exactly as described in ref. Yu et al. (2022). CUT&RUN fragments were sequenced using 79 + 79 nt paired-end reads on a NextSeq500 (Illumina). After mapping raw reads to the mouse genome (mm10) using Bowtie 2.2.5 (ref. Langmead and Salzberg (2012)) with parameters —very-sensitive —no-unal —no-mixed —no-discordant -|10, A-MYB and TCFL5 peaks were identified using Sparse Enrichment Analysis for CUT&RUN (SEARC Meers et al. (2019)) in “norm relaxed” mode. Without antibody was used as the background control.

To be considered bound by TCFL5, a gene was required (1) to have a SEARC-identified peak within 500 bp of its transcription start site in both CUT&RUN replicates; or (2) to have a SEARC-identified peak within 500 bp of its transcription start site in one of the two CUT&RUN replicates and a MACS2-identified peak within 500 bp of its transcription start site in two of three ChIP-seq replicates. To be considered bound by A-MYB, a gene was required (1) to have a SEARC-identified peak within 500 bp of its transcription start site in both CUT&RUN replicates; or (2) to have a SEARC-identified peak within 500 bp of its transcription start site in one of the two CUT&RUN replicates and a MACS2-identified peak within 500 bp of its transcription start site in ChIP-seq. CUT&RUN sequencing statistics are provided in Table S1.

## Results

### TCFL5, a testis-specific transcription factor first expressed at the pachytene stage of meiosis I

**Fig. 1.**
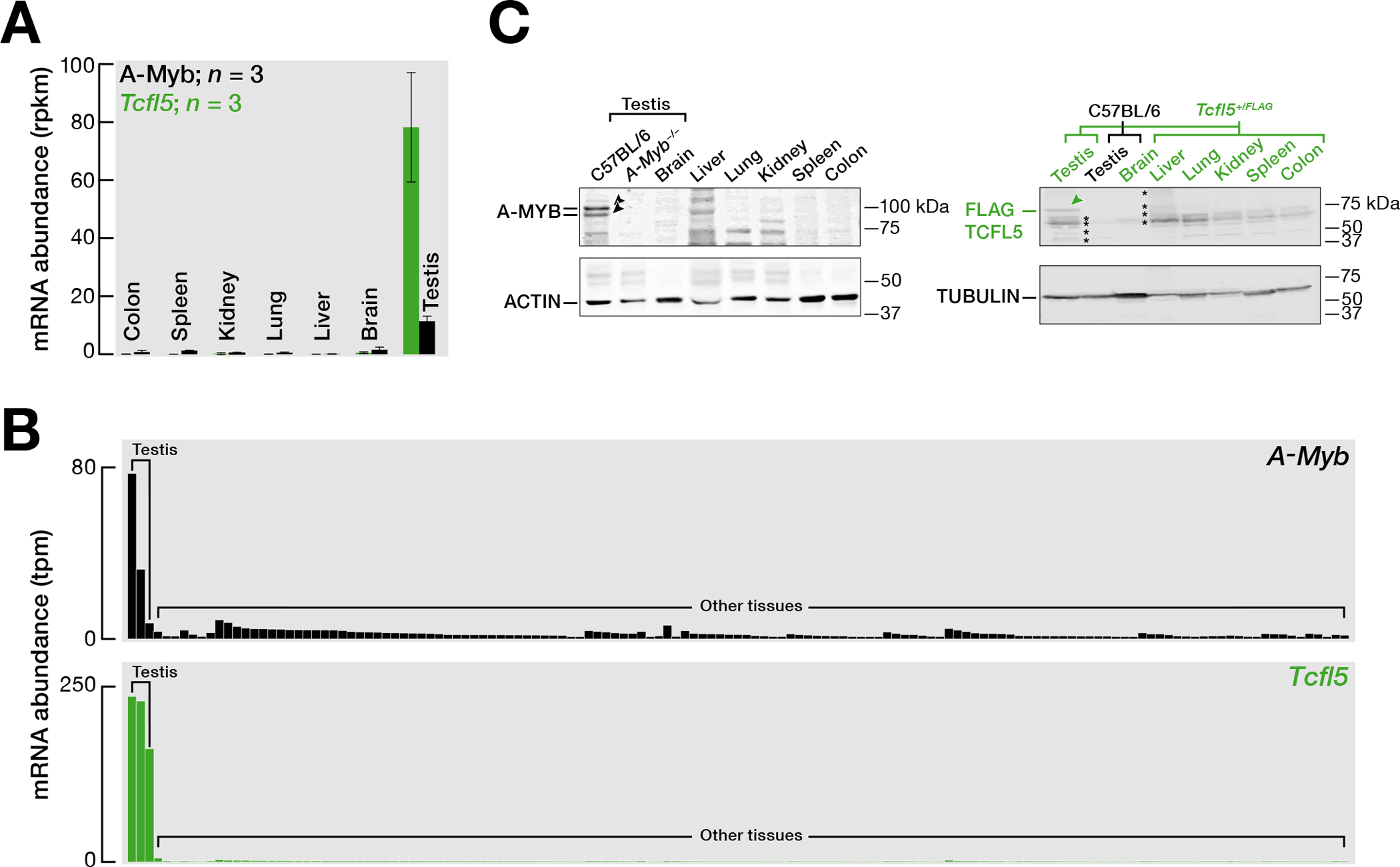
TCFL5 is specifically expressed in primary spermatocytes of testis. **(A)** Steady-state mRNA abundance of *A-Myb* and *Tcfl5* measured by RNA sequencing of various mouse tissues (Merkin et al., 2012). The bar represents the mean mRNA abundance of *A-Myb* and *Tcfl5* from three independent trials. Whiskers show standard error. **(B)** mRNA abundance of *A-Myb* and *Tcfl5* measured by RNA sequencing of various tissues. Data is from the mouse ENCODE project. **(C)** Abundance of A-MYB and FLAG–TCFL5 proteins in various mouse tissues. FLAG-TCFL5 protein was detected in various tissues from *Tcfl5*^*+/FLAG*^ mouse. ACTIN serves as a loading control. Each lane contained 50 μg testis protein. See Fig. S1A for uncropped western blot images. Asterisks represent background from the secondary antibody: See Fig. S1B.

To determine the expression profile of *Tcfl5* mRNA, we used publicly available RNA sequencing data from various mouse tissues (Merkin et al., 2012) (Fig. 1A and 1B); to measure TCFL5 protein abundance, we used *Tcfl5*^*+/FLAG*^ knock-in mice, in which one copy of TCFL5 protein was tagged with an amino terminal 3XFLAG sequence (“FLAG-TCFL5”). FLAG-TCFL5 was detected in testis but not in other tissues we examined (Fig. 1C; Fig. S1A).

Consistent with the testis-specific expression of human *TCFL5* (Maruyama et al., 1998) and the primary spermatocyte-specific expression of mouse *Tcfl5* (Yu et al., 2022), TCFL5 was not detected in purified spermatogonia, was abundant in primary spermatocytes (comprising pachytene and diplotene spermatocytes), and present at a lower level in secondary spermatocytes (Fig. S1C). MILI, which is present in both spermatogonia and spermatocytes, served as a control (Aravin et al., 2006; Aravin et al., 2007; Aravin et al., 2008; Gainetdinov et al., 2018; Kuramochi-Miyagawa et al., 2008; Li et al., 2013; Ozata et al., 2019; Özata et al., 2020).

### STRA8/MEIOSIN initiates a coherent feedforward loop to ensure A-MYB and TCFL5 expression

**Fig. 2.**
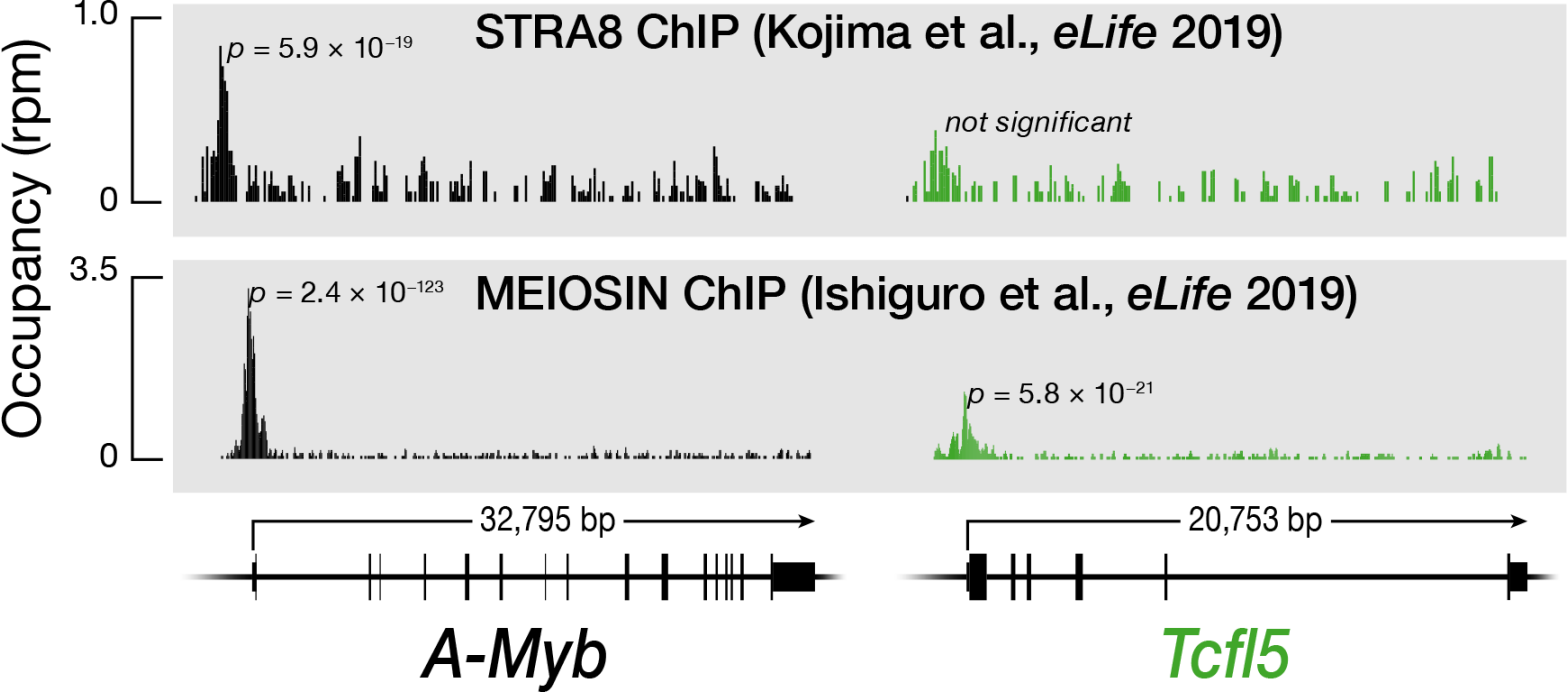
STRA8/MEIOSIN axis initiates the transcription of *A-Myb* and *Tcfl5*. STRA8, MEIOSIN, A-MYB, and TCFL5 ChIP-seq peaks at the promoters of the *A-Myb* and *Tcfl5* genes.

A-MYB promotes its own transcription and directly initiates transcription of *Tcfl5*; TCFL5 responds by reinforcing its own transcription and by increasing the steady-state abundance of A-MYB via positive transcriptional feedback circuit (Li et al., 2013; Yu et al., 2022; Özata et al., 2020). Since the retinoic acid-responsive transcription factor STRA8 and its interacting partner MEIOSIN trigger the entrance of spermatogonia into meiosis I (Ishiguro et al., 2020; Kojima et al., 2019). Our re-analysis of publicly available STRA8 and MEIOSIN ChIP-seq data (Kojima et al., 2019) identified a prominent STRA8 peak within the promoter region of *A-Myb* and prominent MEIOSIN peaks within the promoter regions of both *A-Myb* and *Tcfl5* (Fig. 2). Because *A-Myb* expression precedes that of *Tcfl5* (Yu et al., 2022) and the concentration of retinoic acid drops immediately after entry into meiosis I and remains low until late meiosis I (Endo et al., 2017), we propose that in response to the short burst of retinoic acid, the STRA8/MEIOSIN complex initiates A-MYB expression, whereas TCFL5 production is delayed until A-MYB reaches a concentration threshold. Once the A-MYB concentration reaches its steady-state level, high *Tcfl5* expression persists even after retinoic acid levels decline. This behavior likely reflects the organization of STRA8/MEIOSIN, A-MYB, and TCFL5 into a coherent feed feedforward loop (Alon, 2007). Finally, binding of MEISOIN to the *Tcfl5* promoter suggests that MEIOSIN itself may play a STRA8-independent role in the progression of meiosis, consistent with observations that the pre-leptotene block is more severe in *Meiosin*^*−/−*^ than in *Stra8*^*−/−*^ mice (Ishiguro et al., 2020).

### TCFL5-deficient germ cells arrest later than *A-Myb* mutants

**Fig. 3.**
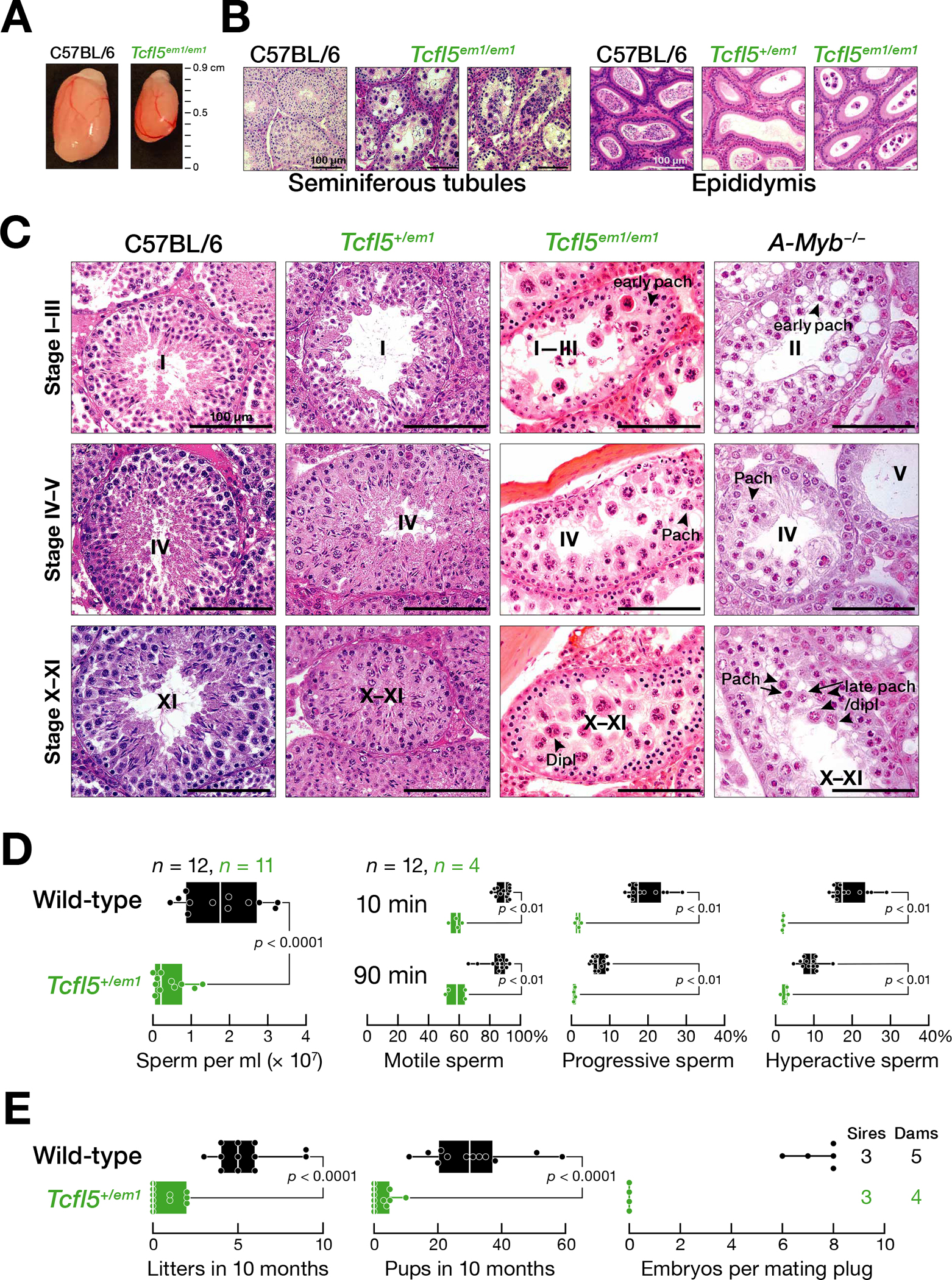
*Tcfl5*^*em1/em1*^ mutant germ cells arrest at the mid-pachytene stage of meiosis I, and *Tcfl5*^*+/em1*^ mutant mice fail to produce functional sperm. **(A)** Comparison of testes from four-month-old C57BL/6 and *Tcfl5*^*em1/em1*^ mice. **(B)** Hematoxylin and eosin staining of testis and epididymis sections from C57BL/6, *Tcfl5*^*em1/em1*^, and *Tcfl5*^*+/em1*^ mice. **(C)** Seminiferous tubules at different epithelial stages from C57BL/6, *Tcfl5*^*em1/em1*^, *Tcfl5*^*+/em1*^, and *A-Myb*^*−/−*^ mice. **(D)** Characterization of sperm from *Tcfl5*^*em1/em1*^ and C57BL/6 caudal epididymis. **(E)** Viable litters and total pups sired in 10 months by two-to-eight-month-old male mice, and the number of embryos present at 14.5 days post-coitus in C57BL/6 females mated to *Tcfl5*^*+/em1*^ or C57BL/6 males. In (**D**) and (**E**), vertical lines denote median, and whiskers mark the minimum and maximum values. Each dot represents an individual male. Significance was measured using a two-tailed unpaired Mann-Whitney U test.

*Tcfl5*^*em1/em1*^ mutant testes lack epididymal sperm (Fig. 3A, B). Consistent with the testis-specific expression of *Tcfl5*, we observed no abnormalities in *Tcfl5*^*em1/em1*^ females. *Tcfl5*^*em1/em1*^ mutant testes arrest spermatogenesis at meiosis I and contain no spermatids or spermatozoa (Fig. 3B). Strikingly, the developing pachytene spermatocytes in *Tcfl5*^*em1/em1*^ mutants formed abnormal multinucleated symplasts (Fig. 3B). These giant cells contain multiple nuclei within a common cytoplasm, suggesting incomplete cytokinesis. TUNEL staining detected no increase in apoptotic germ cells compared to wild-type (Fig. S2A).

Immunostaining to detect the synaptonemal complex protein SYCP3 showed that the chromosomes of the *Tcfl5*^*em1/em1*^ multinucleated pachytene spermatocytes are of normal length and appearance (Fig. S2B, C). In contrast, γH2AX, a marker for double-strand DNA breaks, was inappropriately present on the autosomal chromosomes of ~40% of pachytene spermatocytes of *Tcfl5*^*em1/em1*^ mutant testis (Fig. S2B–D). Similarly, 40% of *A-Myb*^*−/−*^ pachytene spermatocytes featured cloud-like γH2AX staining over the autosomes (Fig. S2C, D), consistent with unrepaired double-stranded DNA breaks persisting in unsynapsed chromatin (Bolcun-Filas et al., 2011). Given that TCFL5 participates in a positive feedback loop that reinforces transcription of *A-Myb*, the presence of γH2AX staining on autosomal chromosomes in *Tcfl5*^*em1/em1*^ likely reflects the reduced abundance of A-MYB in the absence of TCFL5 (Yu et al., 2022).

*A-Myb*^*−/−*^ mutant testis had a loose, defective epithelium by stage II (evidenced by spermatogonial density and size), and some pachytene spermatocytes underwent apoptosis at stages IV and X-XI (Fig. 3C). Compared to stage II tubules, stage IV and stage V tubules contained fewer early pachytene cells. By stage X–XI, few late pachytene or diplotene spermatocytes—judged by their increased size compared to early pachytene cells—were present. In contrast, the epithelium of *Tcfl5*^*em1/em1*^ testes was intact at stage II, and loosening of the attachment of pachytene spermatocytes to the epithelium was not observed before stage IV. The stage XI tubules in *Tcfl5*^*em1/em1*^ mutants contained clusters of three or four diplotene spermatocytes, some of which showed cytoplasm eosin staining, suggesting the onset of apoptosis (Fig. 3C).

### TCFL5 is haploinsufficient

Heterozygous *Tcfl5*^*+/em1*^ testes are not normal. *Tcfl5*^*+/em1*^ have ~5-fold fewer caudal epididymal sperm (two-sided unpaired Mann-Whitney-Wilcoxon U test, *p* < 0.0001) (Fig 3B, D) and fewer motile and progressive sperm. Few sperm were hyperactivated when incubated in capacitating conditions (Fig. 3D). When mated with C57BL/6 females, adult *Tcfl5*^*+/em1*^ males sired fewer viable litters and fewer pups than C57BL/6 males (two-sided unpaired Mann-Whitney-Wilcoxon U test, both *p* < 0.0001) (Fig. 3E). Moreover, the embryos sired by *Tcfl5*^*+/em1*^ males often failed to develop. For example, three *Tcfl5*^*+/em1*^ males were each housed with two C57BL/6 females, and embryos isolated 14.5 days after the appearance of a mating plug. No embryos were detected among the C57BL/6 females paired with *Tcfl5*^*+/em1*^ males (*n* = 4 dams and 3 sires); females paired with C57BL/6 males all carried healthy embryos (7.4 ± 0.9, *n* = 5 dams and 3 sires) (Fig. 3E).

**Fig. 4.**
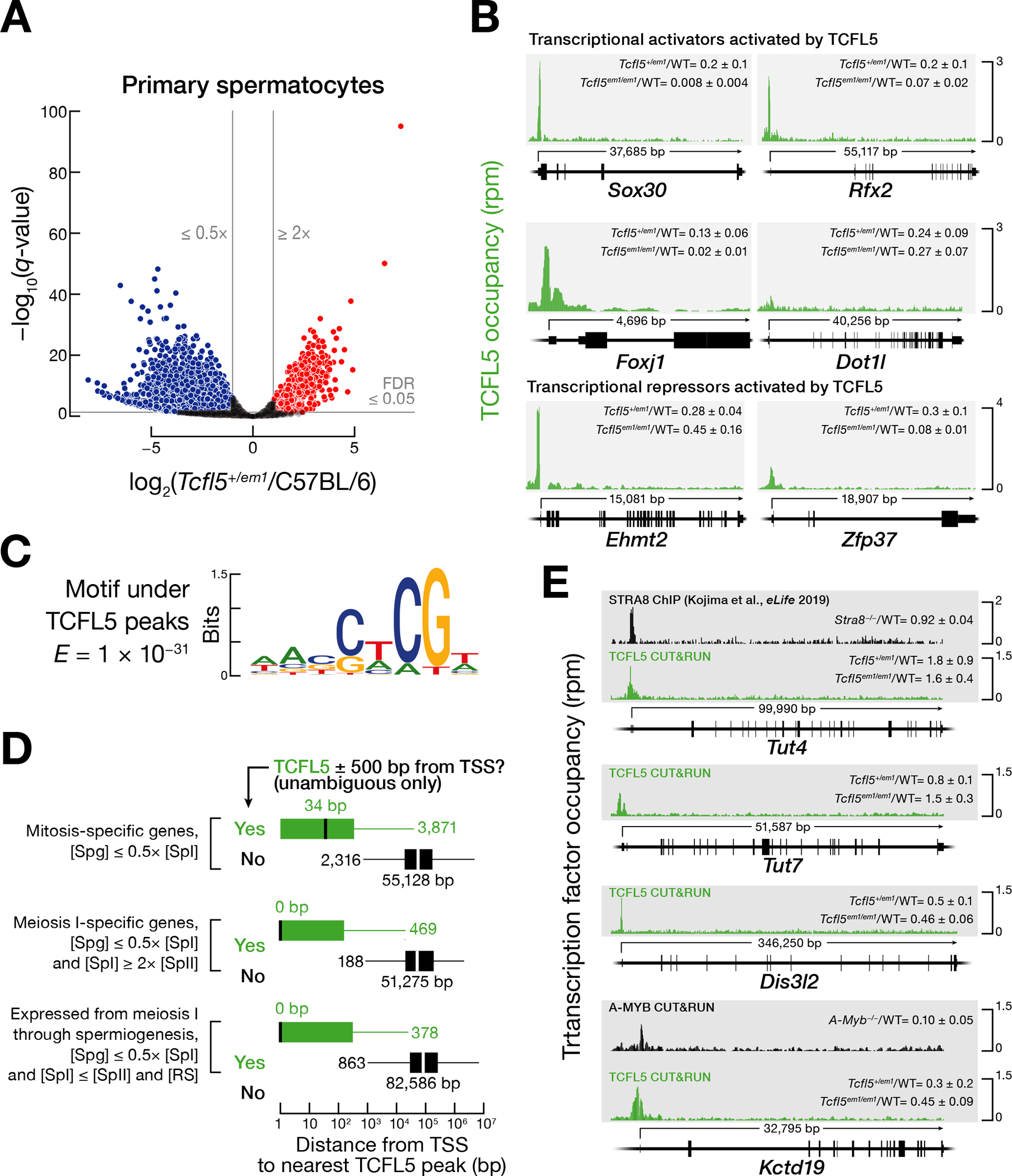
TCFL5 regulates male meiosis I and spermiogenesis. **(A)** The volcano plot shows the genes whose mRNA abundance increased or decreased significantly in FACS-purified primary spermatocytes from *Tcfl5*^*+/em1*^ heterozygotes (*n* = 3), compared to C57BL/6 (*n* = 3). **(B)** TCFL5 CUT&RUN peaks at the promoters of *Sox30*, *Rfx2*, *Foxj1*, *Dot1l*, *Ehmt2*, and *Zfp37*. WT denotes C57BL/6. **(C)** MEME-reported canonical TCFL5 binding motif underlying the TCFL5 CUT&RUN peaks. **(D)** Distance (mean of two trials) from the nearest TCFL5 CUT&RUN peak to the transcription start site (TSS) for three categories of genes: mitosis-specific, meiosis I-specific, and genes whose expression starts at meiosis and persists through spermiogenesis. Genes were classified as not bound by TCFL5 if they had no significant TCFL5 peak ± 500 bp from the transcription start site in any of the CUT&RUN or ChIP-seq datasets. Vertical black lines: median; whiskers: maximum and minimum values, excluding outliers. **(E)** STRA8 ChIP-seq and TCFL5 and A-MYB CUT&RUN peaks at the promoters of *Tut4/7*, *Dis3l2*, and *Kctd19*. WT denotes C57BL/6.

To better understand the underlying molecular defects in *Tcfl5*^*+/em1*^ heterozygotes, we measured steady-state RNA abundance in primary spermatocytes purified from adult testes (Fig. 4A). The mRNA abundance of 4,653 genes in primary spermatocytes were significantly lower in *Tcfl5*^*+/em1*^ compared with C57BL/6 (mutant ÷ control < 0.5 and FDR < 0.05) (Fig. 4A; Table S2A). Notably, the abundance of *Tcfl5* mRNA itself was >5-fold lower in *Tcfl5*^*+/em1*^ primary spermatocytes than in wild-type (mean change = 0.17 ± 0.06). CUT&RUN analysis of TCFL5 DNA binding in wild-type revealed that TCFL5 bound near the transcription start site for 2,173 of 4,653 genes whose transcripts were reduced in *Tcfl5*^*+/em1*^ primary spermatocytes (Fig. S3A).

*Tcfl5* haploinsufficiency made obtaining *Tcfl5*^*em1/em1*^ males daunting: Ten heterozygous breeding pairs produced only 28 pups over five years, of which only four were male homozygotes. Moreover, adult *Tcfl5*^*em1/em1*^ whole testes contain a higher fraction of primary spermatocytes than wild-type, so comparisons of transcript abundance between *Tcfl5*^*em1/em1*^ and wild-type testes underestimate decreases in TCFL5-dependent gene expression.

To further test the idea that TCFL5 is a transcriptional activator, we measured the change in the transcription rate of genes in the absence of one *Tcfl5* allele by performing global-run-on sequencing of FACS-purified primary spermatocytes from *Tcfl5*^*+1/em1*^ heterozygous mutants and C57BL/6 controls. The nascent transcript abundance of 227 genes in primary spermatocytes were significantly lower in *Tcfl5*^*+/em1*^ compared with C57BL/6 (mutant ÷ control < 0.5 and FDR < 0.05) (Fig. S3B; Table S2B). In primary spermatocytes from C57BL/6 controls, the median rate of transcription of genes bound by TCFL5, A-MYB, or both was higher than for genes bound by neither protein (Wilcoxon signed-rank test *p-value* < 2.2 × 10^−16^) (Fig. S3C). CUT&RUN detected TCFL5 at the promoters of 77 of 227 genes whose nascent transcript abundance was reduced in *Tcfl5*^*+/em1*^ primary spermatocytes (Fig. S3D; Table S2B).

### TCFL5 activates transcription of transcription factor genes

Of the 1,304 genes whose steady-state transcript abundance was reduced more than twofold in *Tcfl5*^*em1/em1*^ mutant mice whole testes compared to wild-type (median change = 0.2 ± 0.1) and whose promoters were bound by TCFL5, the transcript abundance of 1,058 genes was also reduced in *Tcfl5*^*+/em1*^ primary spermatocytes (median change = 0.26 ± 0.09). Of these genes, the promoters of only ~42% (443 of 1,058) were occupied by A-MYB, suggesting that >600 genes are transcriptionally activated by TCFL5 rather than A-MYB.

Beyond those genes directly activated by TCFL5, many others are likely regulated by transcription factors that are themselves under TCFL5 control. In fact, 73 chromatin modifying enzymes or sequence-specific, DNA-binding transcription factors whose promoters are bound by TCFL5 showed significantly reduced mRNA abundance in both *Tcfl5*^*+/em1*^ primary spermatocytes and *Tcfl5*^*em1/em1*^ mutant testes compared to wild-type, strong evidence that they require TCFL5 for their activation (Fig. S4A; Table S2A). These include *Sox30*, *Rfx2*, and *Foxj1*, which encode transcription factors likely to participate in spermiogenesis, as well as *Dot1l*, which encodes a conserved methyltransferase that methylates lysine 79 in histone H3 at a subset of enhancer elements and regulates homologous recombination during meiosis in *C. elegans* (Feng et al., 2002; Godfrey et al., 2019; Lascarez-Lagunas et al., 2020) (Fig. 4B). SOX30 is required for male fertility, and both SOX30 and RFX2 activate the expression of genes that participate in spermiogenesis (Feng et al., 2017; Zhang et al., 2018; Wu et al., 2016). The forkhead family transcription factor FOXJ1 activates transcription of ciliary genes (Stubbs et al., 2008; Yu et al., 2008) and regulates sperm motility in frog testes (Beckers et al., 2018). In addition to these transcriptional activators, the 73 TCFL5-regulated transcription factor genes include two repressors, EHMT2 and ZFP37 (Tachibana et al., 2007; Payen et al., 1998) (Fig. 4B). Dimethylation of lysine 9 of histone H3 by EHMT2 has been proposed to ensure that recombination occurs at the correct genomic loci (Tachibana et al., 2007).

### Mapping TCFL5 binding across the genome

We identified genes with high TCFL5 binding occupancy using Sparse Enrichment Analysis for CUT&RUN (SEARC Meers et al. (2019)) to examine CUT&RUN data from FACS-purified primary spermatocytes. SEARC identified 9,290 genes, including 7,664 protein-coding and 332 non-coding genes, with a TCFL5 peak within 500 bp of their transcription start site (Table S3). The sequence motif associated with the CUT&RUN peaks defined the TCFL5 consensus binding site as WANSWCGW (W = A or T; S = G or C; expected value by chance, *E* = 1 × 10^−31^) (Fig. 4C).

To better understand the role of TCFL5 in regulating meiotic gene expression, we classified genes according to their transcript concentration: i.e., molecules per cell corrected for cell volume (Fig. S4B; Table S4A, B). Mitosis-specific genes were defined as those whose transcript concentration in spermatogonia was more than twice that in primary spermatocytes (8,209 genes). Meiosis I-specific genes were required to have a transcript concentration in primary spermatocytes more than twice that in either spermatogonia or secondary spermatocytes (792 genes). mRNAs required during spermiogenesis are mostly transcribed during meiosis (Grunewald et al., 2005; Kierszenbaum and Tres, 1975; Ostermeier et al., 2002). A third category comprised genes turned on at meiosis I and whose mRNAs persisted through spermiogenesis; these were defined as having a transcript concentration in primary spermatocytes that was more than twice that in spermatogonia but was essentially unchanged in secondary spermatocytes and round spermatids (1,466 genes).

The promoters of ~47% of mitosis-specific genes were bound by TCFL5 (3,871 of 8,209 genes; median distance from transcription start site to the nearest TCFL5 peak = 34 bp), suggesting TCFL5 sustains their expression after the onset of meiosis I (Fig. 4D; Table S3). Approximately 59% of the promoters of the meiosis I-specific genes were bound by TCFL5 (469 of 792 genes; median distance from transcription start site to the nearest TCFL5 peak = 0 bp); these genes are likely turned on or up by TCFL5 early in meiosis I. Among the 1,466 genes expressed at meiosis I whose expression persisted through spermiogenesis, TCFL5 bound to the promoters of ~26% (378 of 1,466 genes; median distance from transcription start site to the nearest TCFL5 peak = 0 bp) (Fig. 4D). ChIP-seq of TCFL5 using whole adult testis corroborated the CUT&RUN data from sorted cells (Fig. S4C; Table S3).

Notably, TCFL5 binds the promoters of *Tut4* (*Zcchc11*), *Tut7* (*Zcchc6*), and *Dis3l2* (Fig. 4E). TUT4 and TUT7 are terminal nucleotide transferases that uridylate zygotene-expressed mRNAs whose 3′ untranslated regions contain AU-rich elements (AUUUA; AREs), marking them for destruction by the 3′-to-5′ exoribonuclease DIS3L2 as cells transit from the zygotene to the pachytene stage (Morgan et al., 2019). The mRNA abundance of *Tut7* and *Dis3l2* increased >5-fold in primary spermatocytes, compared to spermatogonia, whereas *Tut4* increased only modestly (~1.6-fold). The expression pattern of *Tut7* and *Dis3l2* suggests that these genes are activated by TCFL5 bound to their promoters. Consistent with this idea, *Dis3l2* mRNA decreased twofold in *Tcfl5*^*em1/em1*^, relative to C57BL/6 (mean decrease = 0.46 ± 0.06 in whole testes and 0.5 ± 0.1 primary spermatocytes) (Fig. 4E). Unexpectedly, *Tut4* mRNA abundance increased ~50% in both *Tcfl5*^em1/em1^ whole testes and *Tcfl5*^*+/em1*^ primary spermatocytes (mean increase = 1.6 ± 0.4 in whole testes and 1.8 ± 0.9 in primary spermatocytes). Although we cannot exclude the possibility that TCFL5 represses expression of *Tut4* but activates *Dis3l2*, we propose an alternative explanation. TCFL5 may activate the expression of all three, but the expected decrease of *Tut4* and *Tut7* mRNA in *Tcfl5* mutants is obscured by an increase in their mRNA stability because *Tut4* and *Tut7* mRNAs are themselves ARE-dependent targets of TUT4/TUT7-catalyzed uridylation and DIS3L2 degradation. In this view, the decrease in *Dis3l2* expression in *Tcfl5* mutants causes *Tut4* and *Tut7* mRNAs to become more stable. Supporting this view, the *Tut4* 3′ UTR contains three canonical AREs, and the 1030-nt long *Tut7* 3′ UTR both contains one canonical ARE and is longer than typical for testis (median = 639 nt), a general feature of TUT4/7 targets (median = 1,703 nt) (Morgan et al., 2019).

Finally, the set of promoters bound by TCFL5 includes *Kctd19* (Fig. 4E), a gene whose protein product is essential for meiotic exit (Horisawa-Takada et al., 2021; Oura et al., 2021). The *Kctd19* promoter is also bound by A-MYB. The abundance of *Kctd19* mRNA increases >200-fold when spermatogonia differentiate into spermatocytes (Table S4A). *Kctd19* mRNA abundance declined >3-fold in *Tcfl5*^*+1/em1*^ and 10-fold in *A-Myb*^*−/−*^ primary spermatocytes.

### A-MYB/TCFL5 regulatory architecture establishes a coherent feedforward loop to amplify miR-34/449 production

**Fig. 5.**
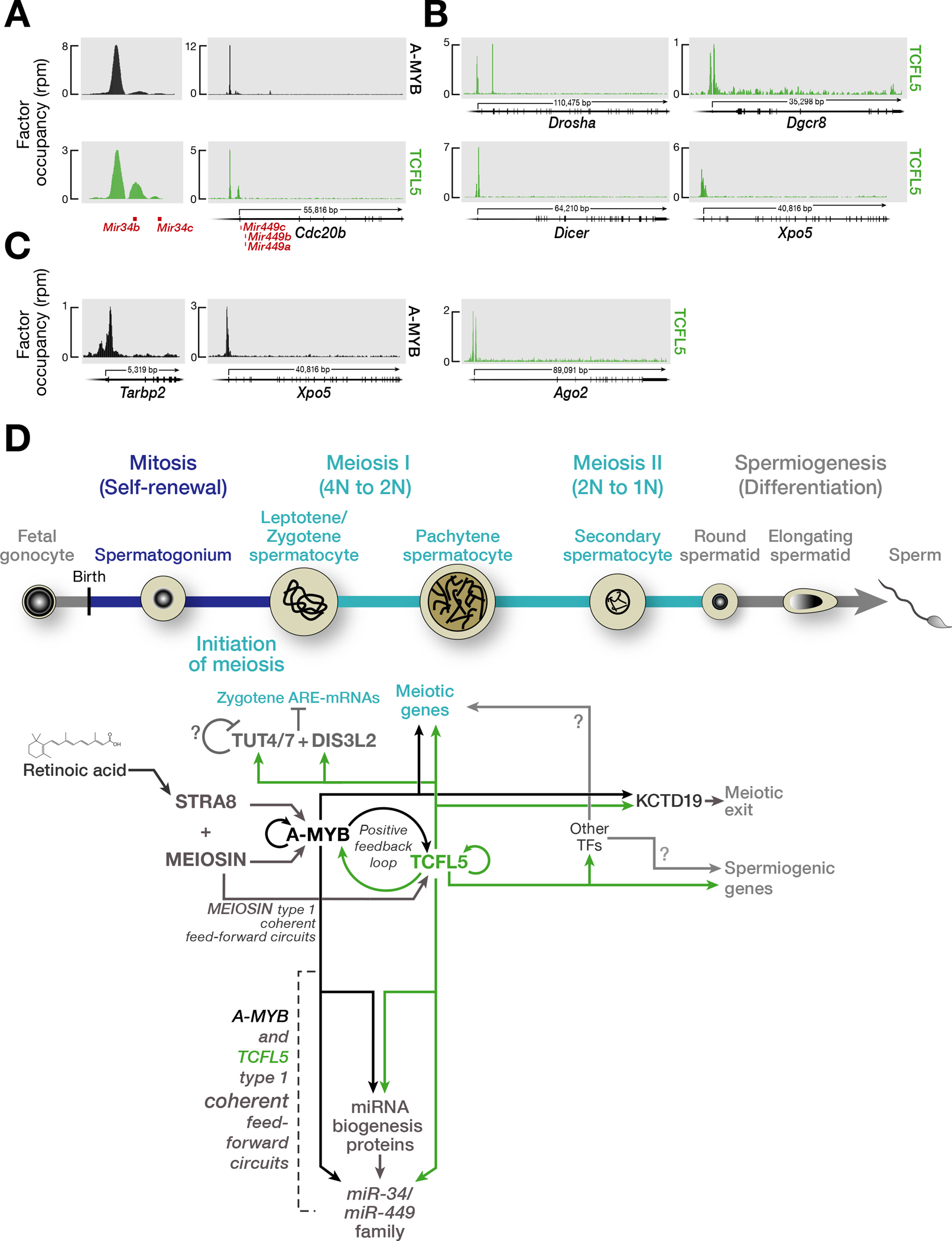
A-MYB/TCFL5 regulatory architecture establishes coherent feedforward loop to burst the expression of *miR34/miR449* family, and a model for the transcriptional architecture of mouse male meiotic cells. **(A)** A-MYB and TCFL5 CUT&RUN peaks at the promoters of *miR-34b/c* and *miR-449a/b/c* genes. **(B,C)** A-MYB and TCFL5 occupancies around the promoters of genes encoding miRNA maturation proteins: TCFL5 occupancy **(B)** A-MYB occupancy **(C).** **(D)** The model incorporates hypotheses from refs. (Bolcun-Filas et al., 2011; Galán-Martínez et al., 2022; Horisawa-Takada et al., 2021; Ishiguro et al., 2020; Kojima et al., 2019; Oura et al., 2021; Xu et al., 2022; Yu et al., 2022) and this study. The developmental progression of spermatogenesis is aligned with the time of expression of various proteins. The figure highlights the proposed sequential roles of STRA8, MEIOSIN, A-MYB, and TCFL5, calling attention to the underlying architecture of the transcriptional circuits regulating male meiosis in mouse.

The miR-34/449 miRNA family comprises two highly conserved clusters, *miR-34a/b/c* and *miR-449a/b/c*, whose miRNAs share a single seed sequence, the miRNA region that determines target specificity. The miR-34/449 family is essential for spermatogenesis and for ciliogenesis of efferent ductules epithelium (Bao et al., 2012; Comazzetto et al., 2014; Marcet et al., 2011; Song et al., 2014; Wu et al., 2014). While *miR-34a* (chromosome 4) is detectable at low levels in spermatogonia and through later stages of spermatogenesis(Bao et al., 2012), the expression of *miR-34b/c* and *miR-449a/b/c* increases >10-fold at the onset of meiosis I (Comazzetto et al., 2014). Our data identified prominent TCFL5 peaks on the promoters of the *miR-34b* and *miR-34c* genes; A-MYB bound to the promoter of the *miR-34b* locus only (Fig. 5A). The *miR-449a/b/c* cluster resides in the second intron of *Cdc20b* gene, and both A-MYB and TCFL5 bound to the promoter of *Cdc20b* (Fig. 5A). By contrast, we detect no A-MYB nor TCFL5 binding around the promoter of *miR-34a*. Finally, we detected TCFL5 peaks near the transcription start sites of the promoters of five essential miRNA biogenesis genes: *Drosha*, *Dgcr8*, *Dicer*, *Xpo5*, and *Ago2*, significantly more than expected by chance by comparison to protein-coding genes generally (Chi-square test *p* = < 2.0 × 10^−3^) (Fig. 5B). A-MYB bound to the promoters of *Xpo5* and *Tarbp2*, a Dicer partner protein (Fig. 5C). In summary, the A-MYB/TCFL5 regulatory architecture activates the transcription of miRNA genes essential for spermatogenesis and genes encoding miRNA biogenesis proteins, thereby generating a feedforward loop predicted to ensure accumulation of miR-34/miR-449 family miRNAs during meiosis I.

### The regulatory architecture that drives miR-34/449 production in rodents is conserved in rhesus macaque

We recently demonstrated that TCFL5 orthologs are present in the genomes of amniotes (Yu et al., 2022). We then tested whether TCFL5 and A-MYB similarly organize a coherent feedforward loop that ensures the robust production of MIR34/449 in rhesus macaque. A-MYB and TCFL5 ChIP-seq identified significant peaks at the promoters of *MIR34B/*C and *CDC20B,* whose second intron hosts *MIR449A/B* (Fig. S5A). Furthermore, the promoters of five of the six key miRNA biogenesis genes featured a TCFL5 or an A-MYB peak near their transcription start sites (*DROSHA*, *DGCR8*, *DICER*, *TARBP2*, and *XPO5;* Chi-square test *p* = < 2.0 × 10^−3^) (Fig. S5B). Together, these data suggest that the regulation miR-34/449 miRNA family by A-MYB/TCFL5 transcriptional architecture was found in the last common ancestor of Glires and primates.

## DISCUSSION

Our data, together with those from previous studies (Bolcun-Filas et al., 2011; Galán-Martínez et al., 2022; Horisawa-Takada et al., 2021; Ishiguro et al., 2020; Kojima et al., 2019; Oura et al., 2021; Xu et al., 2022; Yu et al., 2022), suggest a model for the evolutionarily conserved transcriptional architecture by which A-MYB and TCFL5 collaborate to reprogram gene expression at the onset of male meiosis I (Fig. 5D). Immediately before meiosis begins, a burst of retinoic acid induces transcription of *Stra8* and *Meiosin*, whose protein products play essential roles in the initiation of meiosis I. STRA8/MEIOSIN, in turn, activates *A-Myb* transcription during the leptotene or zygotene phases of meiotic prophase I, a period characterized by general transcriptional quiescence. By the onset of the pachynema, A-MYB initiates transcription of *Tcfl5*, and A-MYB and TCFL5 mutually reinforce their own transcription via interlocking positive feedback loops (Yu et al., 2022). Together A-MYB and TCFL5 directly promote transcription of hundreds of genes encoding proteins required for meiosis and spermiogenesis. These include components of the miRNA biogenesis machinery and the *miR-34/449* miRNA family, promoting the accumulation of miR-34/449 family miRNAs.

Although STRA8 is expressed in early and intermediate spermatogonia, it does not suffice to initiate meiosis in these cells (Endo et al., 2015; Zhou et al., 2008). STRA8 requires its partner protein MEIOSIN to drive the transcription of genes required for meiotic initiation and progression—including *A-Myb* (Ishiguro et al., 2020) (Fig. 2). Our re-analysis of publicly available STRA8 (Kojima et al., 2019) and MEIOSIN ChIP-seq data (Ishiguro et al., 2020) revealed that only MEIOSIN binds the *Tclfl5* promoter, suggesting that MEIOSIN can both collaborate with STRA8 and function independently.

What mechanisms underlie the reduced fertility phenotype observed in the absence of a single allele of *Tcfl5*? TCFL5 is a basic Helix-Loop-Helix (bHLH) transcription factor (Siep et al., 2004). bHLH transcription factors often regulate gene expression in a tissue-specific manner (Bhattacharya and Baker, 2011) and require interacting partners to function (Bhattacharya and Baker, 2011; Shively et al., 2019). Could then TCFL5 require a co-factor? Does reduced intracellular concentration of TCFL5 compromise the function of its co-factor in *Tcfl5*^*+/em1*^ mutant mice? However, the tissue-specific activity of bHLH transcription factors can be conferred by the aid of broadly expressed co-factors, whose expression threshold can be the rate limiting factor for activity of bHLH transcription factors. Along the parallel lines, expression of possible co-factor might be directly regulated by TCFL5. Halving the intracellular concentration of TCFL5 in *Tcfl5*^*+/em1*^ mutant mice may therefore result in amplified negative effect on TCFL5 function itself.

## Supplementary Material

01

02

03

04

05

06

07

08

09

10

## Statistical analyses

All statistics were calculated using R console (https://www.rstudio.com); graphs were generated using R console or Prism 8.4.3 (GraphPad Software, LLC.). For box plots, boxes represent the first and third quartiles, except in Figure 3, where whiskers show the maximum and minimum values excluding outliers. Outliers are defined as data points with values > third quartile + 1.5 × IQR or lower than the first quartile − 1.5 × IQR, where IQR (interquartile range) is the maximum third quartile minus the first quartile minimum. Two-sided Mann-Whitney-Wilcoxon U test was used to calculate *p-value*s. *q*-values in Figure 4A correspond to Benjamini-Hochberg FDR-corrected *p*-values
