# Supplementary material for: The transcription factor TCFL5 responds to A-MYB to elaborate the male meiotic program in mice
Source: Reproduction. Author manuscript; Available in PMC 2023 Feb 1. (PMC9812935; doi:10.1530/REP-22-0355)
Supplement: 01 [file NIHMS1854436-supplement-01.docx]

Supplementary Materials for

**The testis-specific transcription factor TCFL5 responds to A‑MYB to elaborate the male meiotic program in mice**

Katharine Cecchini, Tianxiong Yu, Adriano Biasini, Martin Säflund, Haiwei Mou, Amena Arif, Atiyeh Eghbali, Cansu Colpan, Ildar Gainetdinov, Dirk G. de Rooij, Zhiping Weng, Phillip D. Zamore* and Deniz M. Özata*

*Corresponding author. Email: phillip.zamore@umassmed.edu and deniz.ozata@su.se

**Fig. S1.**

Temporal and spatial expression of A-MYB and TCFL5.

**(A)** Protein abundance of A-MYB and TCFL5 in various tissues (50 µg testis protein per lane). ACTIN serves as a loading control.

**(B)** Blot representing the background from the goat anti-mouse IRDye 800CW secondary antibody.

**(D)** FACS-purified germ cells from C57BL/6 mice (protein from ~100,000 germ cells per lane).

Fig. S2.

**DNA double strand breaks (DBSs) are repaired in *Tcfl5^em1/em1^* but not *A-Myb^−/−^*** **mice.**

**(A)** Terminal deoxynucleotide transferase dUTP nick end-labeling (TUNEL) assay was used to detect apoptotic cells. C57BL/6 tubules treated with 0.5 U DNase served as a positive control for DNA breaks.

**(B)** Immunofluorescent staining was used to detect Synaptonemal Complex Protein 3 (SYCP3) and γH2AX proteins in cryopreserved C57BL/6 and *Tcfl5^em1/em1^* mouse testes sections.

**(C)** Immunostaining to detect SYCP3 and γH2AX proteins on chromosome spreads of spermatocyte nuclei. DAPI was used to stain double-stranded DNA. Representative images are shown.

**(D)** Quantification of γH2AX staining of chromosomes for the chromosome spread analysis shown in (**C**). *N* indicates the number of nuclei examined.

Fig. S3.

**Steady-state transcript abundance and transcription rate of genes bound by TCFL5 is reduced in *Tcfl5^+/em1^*** mice.

**(A)** Boxplots display the mean distance of two replicates from the annotated transcription start site (TSS) to the nearest TCFL5 peak determined by CUT&RUN for genes whose transcripts were significantly reduced in primary spermatocytes purified by FACS from *Tcfl5^+/em1^* testes. Genes classified as not bound by TCFL5 showed no TCFL5 peak ±500 bp from the TSS in any CUT&RUN or ChIP-seq datasets. Vertical lines: median. Whiskers: maximum and minimum values, excluding outliers (i.e., 1.5 × IQR). Measurements with the same values are indicated by a single marker indicating number of individual data points.

**(B)** The volcano plot shows the genes whose transcription rate increased or decreased significantly in FACS-purified primary spermatocytes from *Tcfl5^+/em1^* heterozygotes (*n* = 3), compared to C57BL/6 (*n* = 3).

**(C)** Global-run-on sequencing read density on genes classified as TCFL5-bound, A-MYB-bound, bound by both, and bound by the neither of the factors in C57BL/6 mice.

**(D)** Boxplots display the mean distance of two replicates from the annotated TSS to the nearest TCFL5 peak determined by CUT&RUN for genes whose transcription rate were significantly reduced in primary spermatocytes purified by FACS from *Tcfl5^+/em1^* testes.

**Fig. S4.**

**TCFL5 regulates genes required for meiosis and spermiogenesis and genes encoding downstream transcription factors.**

**(A)** Scatter plot of the steady-state mRNA abundance for 73 TCFL5-bound, transcription factor-encoding genes in *Tcfl5^+/em1^* primary spermatocytes or *Tcfl5^em1/em1^* whole testes compared to C57BL/6 controls. Data points correspond to the mean of three C57BL/6 and three *Tcfl5^+1/em1^* trials (top panel) or six C57BL/6 and two *Tcfl5^em1/em1^* trials.

**(B)** Box plots show transcript concentration (i.e., abundance normalized to cell volume) for three classes of genes in spermatogonia (Spg), primary (SpI) and secondary (SpII) spermatocytes, and round spermatids (RS). Vertical lines: median; whiskers: maximum and minimum values, excluding outliers (i.e., 1.5 × IQR).

**(C)** The distance (mean of three replicates) from the nearest TCFL5 peak, detected by ChIP-seq, to the transcription start site (TSS) for three classes of genes. Genes were only scored as not bound by TCFL5 if they had no TCFL5 peak ±500 bp from the TSS in any CUT&RUN or ChIP-seq datasets. Vertical lines: median. Whiskers: maximum and minimum values, excluding outliers (i.e., 1.5 × **Fig. S5.**

**The production of miR34/miR449 by A-MYB/TCFL5-driven coherent feedforward loop is conserved in rhesus macaque.**

**(A, B)** A‑MYB and TCFL5 ChIP-seq peaks at the promoters of the rhesus macaque *MIR34B/C* and *MIR449A/B* genes **(A)** genes encoding miRNA maturation proteins **(B)**

**Table S1.**

**High-throughput sequencing statistics.**Table S2.

**Genes dysregulated in *Tcfl5^+/em1^* primary spermatocytes.**

DESeq2 analysis shows the list of genes whose steady-state **(A)** and nascent **(B)** transcript abundance declined significantly in *Tcfl5^+/em1^* primary spermatocytes compared to C57BL/6.

**Table S3.**

**Genes regulated by TCFL5.**

TCFL5 CUT&RUN and ChIP-seq analysis. The table reports the signal of the peak for each gene and the distance between the TSS and the nearest TCFL5 peak. Transcript abundance is reported in RPKM for *Tcfl5^em1/em1^* and C57BL/6 mice.

Table S4.

**Three classes of differentially expressed genes during spermatogenesis.**

**(A)** Absolute transcript abundance normalized to cell volume of all genes in FACS-purified C57BL/6 mouse in spermatogonia (Spg), primary spermatocytes (SpI), secondary spermatocyte (SpII), and round spermatids (RS).

**(B)** Mitosis-specific genes, meiosis I-specific genes, and genes that are turned on during meiosis I and whose expression persists through spermiogenesis.
