# Supplementary figures and images for: The transcription factor TCFL5 responds to A-MYB to elaborate the male meiotic program in mice
Source: Reproduction. Author manuscript; Available in PMC 2023 Feb 1. (PMC9812935; doi:10.1530/REP-22-0355)

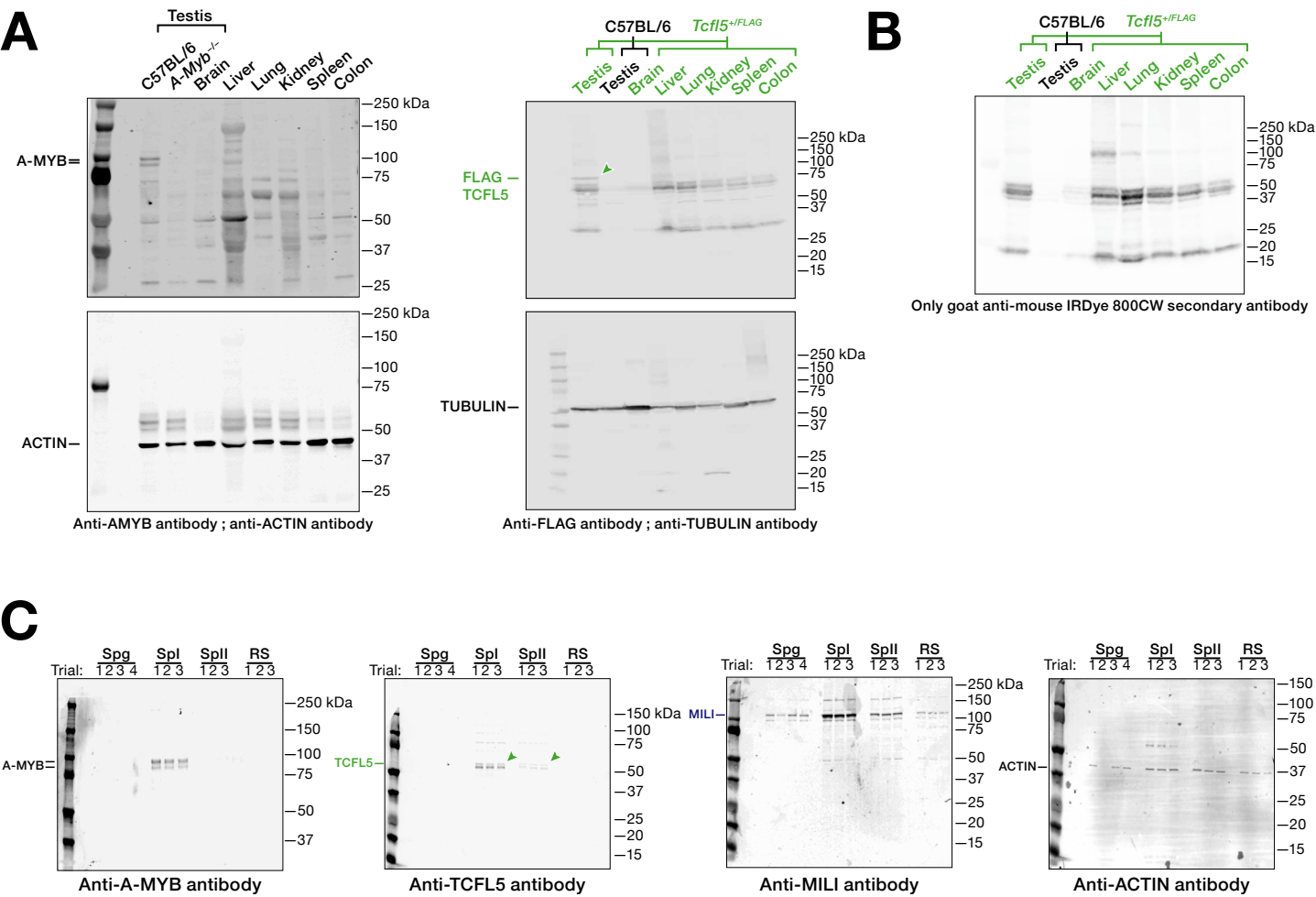

Supplement: 02 [file NIHMS1854436-supplement-02.pdf]

**A**

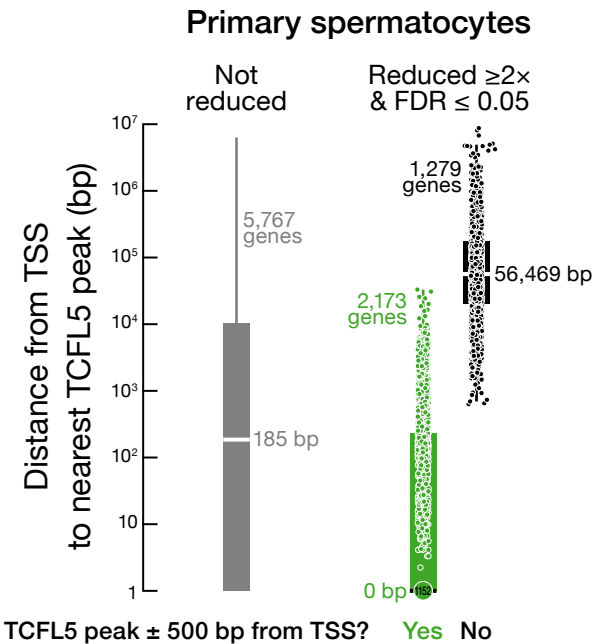

**B**

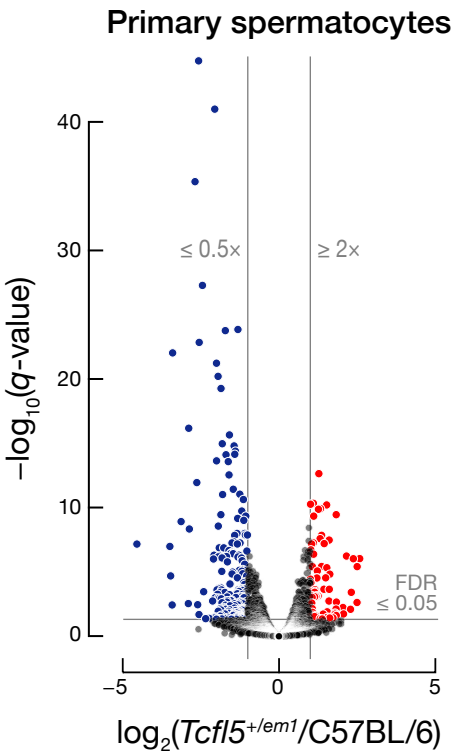

**C**

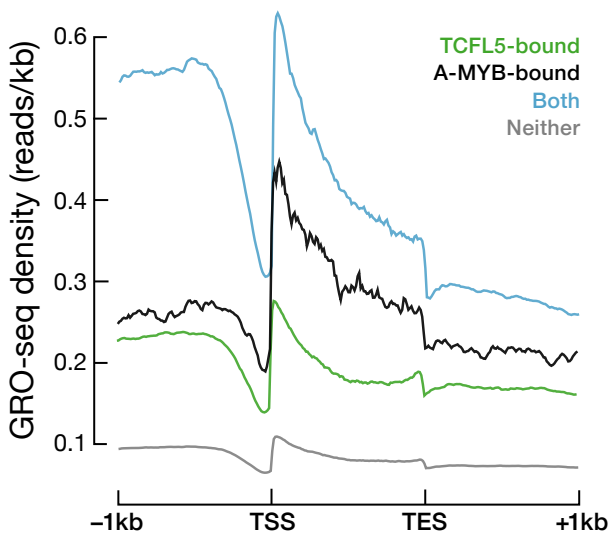

**D**

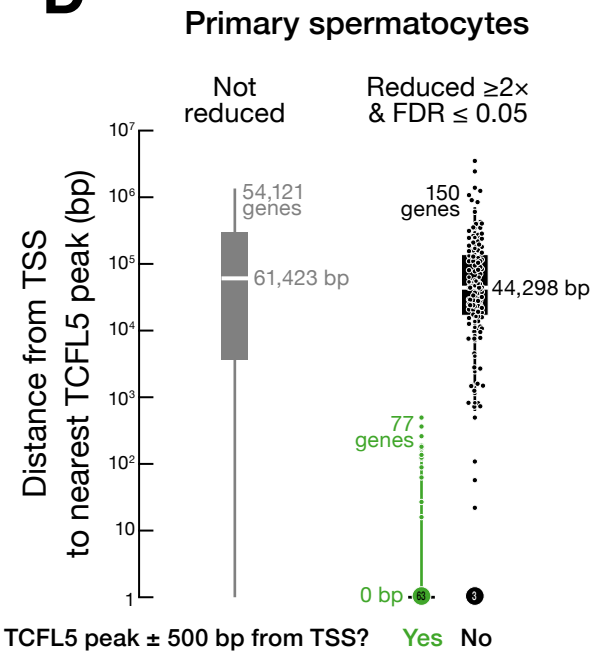

Supplement: 04 [file NIHMS1854436-supplement-04.pdf]

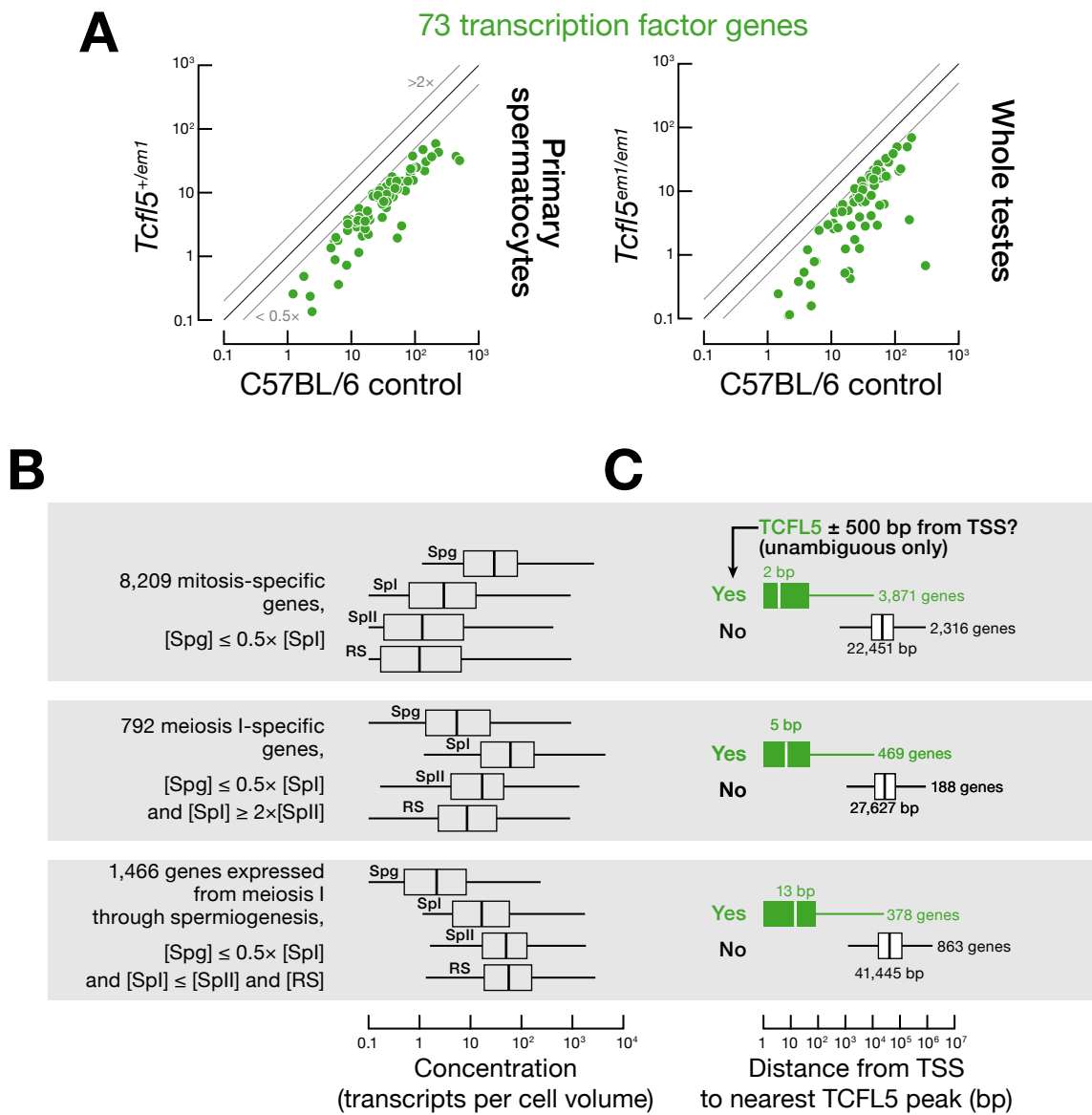

Supplement: 05 [file NIHMS1854436-supplement-05.pdf]

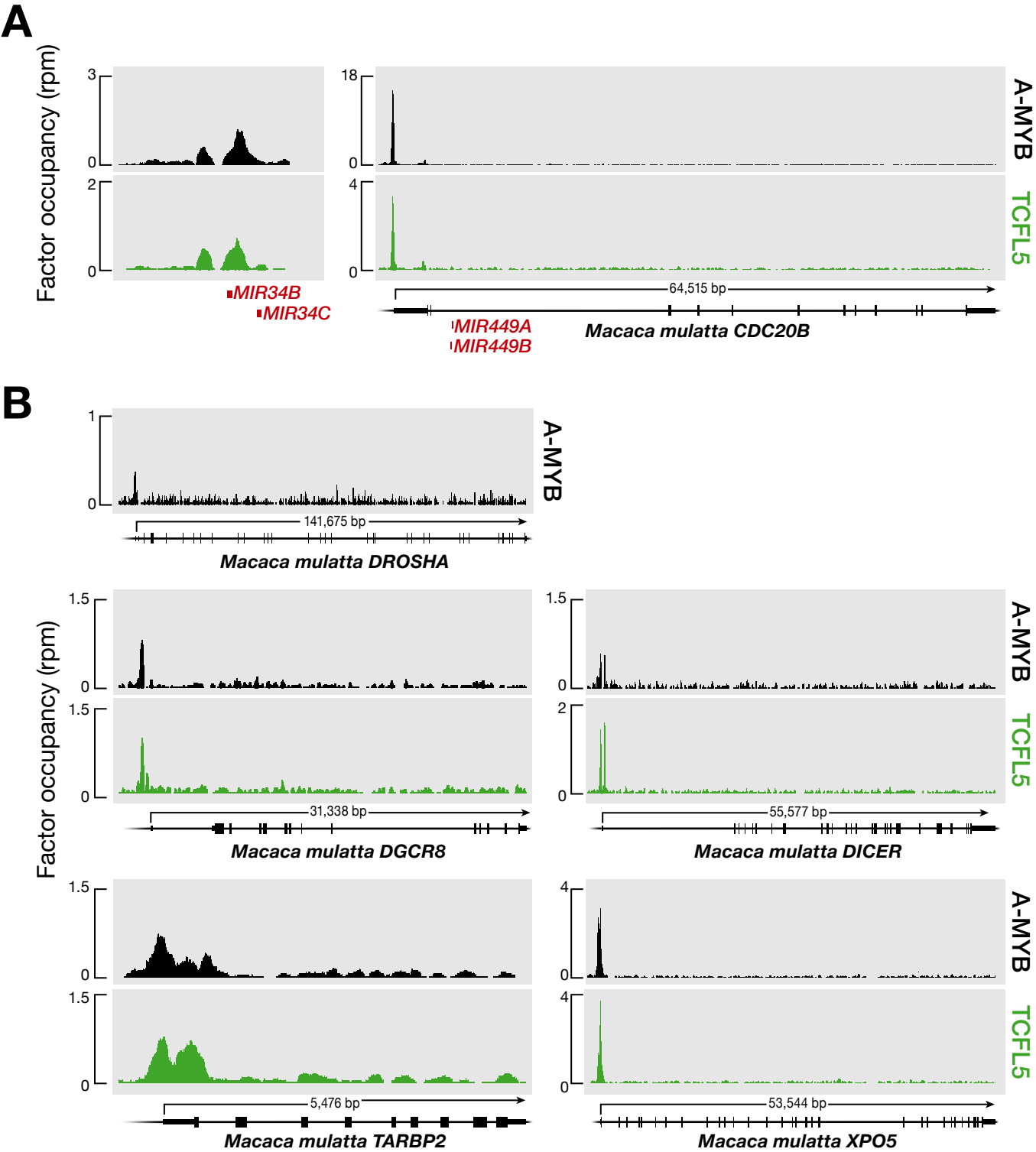

Supplement: 06 [file NIHMS1854436-supplement-06.pdf]
