# Supplementary material for: The transcription factor TCFL5 responds to A-MYB to elaborate the male meiotic program in mice
Source: Reproduction. Author manuscript; Available in PMC 2023 Feb 1. (PMC9812935; doi:10.1530/REP-22-0355)
Supplement: 03 [file NIHMS1854436-supplement-03.pdf]

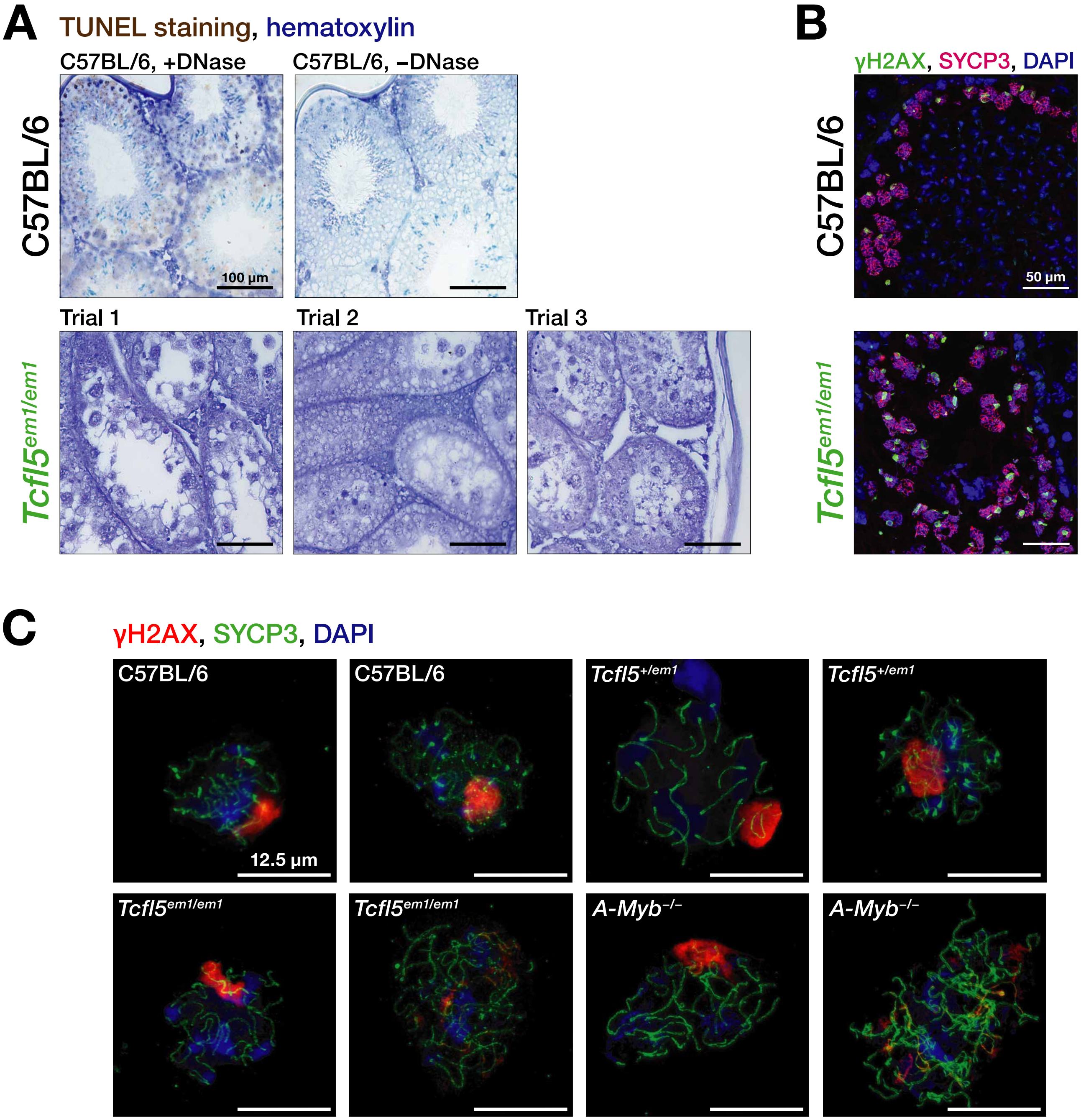

D

	Cells with γH2AX on sex body only	Cells with γH2AX on both autosomes and sex body	Cells with γH2AX but no sex body	*N*
C57BL/6	132 (97%)	4 (3%)	0	136
Tcfl5$^{+/em1}$	102 (85%)	13 (11%)	5 (4%)	120
Tcfl5$^{em1/em1}$	65 (59%)	41 (37%)	4 (4%)	110
A-Myb$^{-/-}$	73 (60%)	32 (26%)	17 (14%)	122
